# Sex differences in adult mood and in stress-induced transcriptional coherence across mesocorticolimbic circuitry

**DOI:** 10.1038/s41398-020-0742-9

**Published:** 2020-02-06

**Authors:** William Paden, Kelly Barko, Rachel Puralewski, Kelly M. Cahill, Zhiguang Huo, Micah A. Shelton, George C. Tseng, Ryan W. Logan, Marianne L. Seney

**Affiliations:** 1 Department of Psychiatry, University of Pittsburgh School of Medicine, Pittsburgh, PA USA; 2 Translational Neuroscience Program, University of Pittsburgh School of Medicine, Pittsburgh, PA USA; 3 Department of Biostatistics, Graduate School of Public Health, University of Pittsburgh, Pittsburgh, PA 15261 USA; 4 Department of Biostatistics, University of Florida, Gainesville, FL 32611 USA; 5 Department of Computational and Systems Biology, University of Pittsburgh School of Medicine, Pittsburgh, PA 15213 USA; 6 Center for Systems Neurogenetics of Addiction, The Jackson Laboratory, Bar Harbor, ME 14609 USA

**Keywords:** Psychiatric disorders, Molecular neuroscience

## Abstract

Women are approximately two times as likely to be diagnosed with major depressive disorder (MDD) compared to men. While sex differences in MDD might be driven by circulating gonadal hormones, we hypothesized that developmental hormone exposure and/or genetic sex might play a role. Mice were gonadectomized in adulthood to isolate the role of developmental hormones. We examined the effects of developmental gonadal and genetic sex on anhedonia-/depressive-like behaviors under non-stress and chronic stress conditions and performed RNA-sequencing in three mood-relevant brain regions. We used an integrative network approach to identify transcriptional modules and stress-specific hub genes regulating stress susceptibility, with a focus on whether these differed by sex. After identifying sex differences in anhedonia-/depressive-like behaviors (female > male), we show that both developmental hormone exposure (gonadal female > gonadal male) and genetic sex (XX > XY) contribute to the sex difference. The top biological pathways represented by differentially expressed genes were related to immune function; we identify which differentially expressed genes are driven by developmental gonadal or genetic sex. There was very little overlap in genes affected by chronic stress in males and females. We also identified highly co-expressed gene modules affected by stress, some of which were affected in opposite directions in males and females. Since all mice had equivalent hormone exposure in adulthood, these results suggest that sex differences in gonadal hormone exposure during sensitive developmental periods program adult sex differences in mood, and that these sex differences are independent of adult circulating gonadal hormones.

## Introduction

Major depressive disorder (MDD) is a debilitating disease affecting ~6.7% of the US population and a leading cause of disability worldwide^1^. Symptoms of MDD include emotional dysregulation, cognitive deficits, and anhedonia. Anhedonia, the reduced ability to experience pleasure and lack of interest in once “rewarding” activities, is present in ~37% of MDD patients, and often predicts poor treatment response^2^. The mesocorticolimbic system is a primary circuit of emotion and mood regulation, comprised of the prefrontal and anterior cingulate cortices, hippocampus, amygdala, dorsal striatum, and nucleus accumbens (NAc), along with other regions^3^. Evidence suggests dysfunction of the mesocorticolimbic system in MDD [e.g., refs. ^4–6^]. For example, neuroimaging studies reveal that individuals with MDD have sustained amygdala hyperactivity, altered prefrontal cortex (PFC) functionality, and reduced activity of the NAc in response to rewarding stimuli^7–9^.

Women are twice as likely to be diagnosed with MDD and often report higher frequency of symptoms and increased symptom severity^10–12^. These sex differences in MDD might be driven by gonadal hormone differences between men and women (i.e., transient activational hormone effects). Indeed, both female and male gonadal hormones influence mood in humans (e.g., premenstrual dysphoric disorder in women^13,14^ and low testosterone in men^15,16^) and in animals (e.g., refs. ^17,18^). However, MDD prevalence remains higher in women across life stages and hormonal states^19,20^, suggesting that other sex-related factors contribute to higher rates of MDD in women. These sex differences could be driven by (1) permanent organizational effects of gonadal hormones during sensitive developmental periods and/or (2) effects of genetic sex^21,22^. Independent investigation of these factors is not possible using wild-type mice, as genetic sex (XX vs. XY) determines gonadal sex (ovaries vs. testes). However, by using the Four Core genotypes (FCG) mice, the contribution of genetic sex (XX/XY) and gonadal sex (ovaries/testes) can be independently investigated^23^. In FCG mice, the testes-determining *Sry* gene was spontaneously deleted from the Y chromosome and placed back onto an autosome. Thus, in these mice, the contribution of these sex-related factors to behavior can be independently investigated. Since we were particularly focused on the effects of developmental gonadal sex, we gonadectomized (GDX) mice to remove the potential confounding activational effects of adult circulating gonadal hormones. Using FCG mice, we previously found an effect of genetic sex on anxiety-like behavior^17^.

To understand the contribution of gonadal sex and genetic sex to depressive-/anhedonia-like behavior and related transcriptional changes, we utilized a chronic stress exposure, behavior testing, and RNA-sequencing (RNA-seq) of several major nodes in the mesocorticolimbic circuit. We found sex differences in depressive-/anhedonia-like behavior were driven by both developmental hormone exposure and genetic sex. We also identified potential candidate genes underlying these behavioral differences and discovered gene modules affected by stress, some of which are altered in opposite directions in males and females. Network analysis predicted potential upstream regulators of these sex-specific effects on gene expression patterns. Together, these data provide new insights into how chronic stress differentially affects males and females.

## Materials and methods

### Mice

Mice were group-housed (3–5 mice/cage) and maintained under standard conditions (12:12 h light/dark cycle; lights on 7 a.m.; 22 ± 1 °C, food and water ad libitum), in accordance with University of Pittsburgh Institutional Animal Care and Use Committee. FCG mice (C57BL/6J background) from our in-house colony were used for all studies; these mice were originally obtained from The Jackson Laboratory (Bar Harbor, ME). We indicate both genetic and gonadal sex for each genotype: (1) XX = XX Female; (2) XY^−^ = XY Female; (3) XX*Sry* = XX Male; (4) XY^−^*Sry* = XY Male.

### Experimental design

**Fig. 1:**
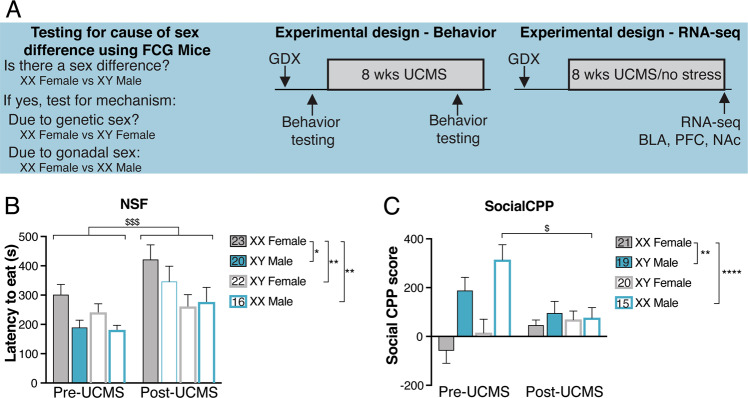
Developmental hormone exposure programs adult sex differences in anhedonia-/depressive-like behavior. **a** Schematic detailing the FCG mouse model and the experimental approach. In FCG mice, we can independently examine the effects of developmental gonadal and genetic sex on mood-related behavior and gene expression. **b** In the NSF, the two-way repeated measures ANOVA indicated the main effects of genotype (*p* < 0.05) and time (*p* < 0.001). The sex difference in latency to eat (*p* < 0.05) was recapitulated by gonadal sex (*p* < 0.01) and by genetic sex (*p* < 0.01). **c** In the social CPP test of social reward, the two-way repeated measures ANOVA indicated a main effect of genotype (*p* < 0.001) and an interaction of genotype and time (*p* < 0.01). The sex difference in social CPP score (*p* < 0.01) was recapitulated by gonadal sex (*p* < 0.0001). Post hoc analyses indicated that stress decreased social CPP score only in XX Males (*p* < 0.05). The number of mice for each genotype is indicated in the legend for each behavioral test. Effect of genotype: *p < 0.05, **p < 0.01; ****p < 0.0001. Effect of stress: ^$^*p* < 0.01; ^$$$^*p* < 0.001.

We tested the effects of genetic sex and gonadal sex on adult anhedonia-/depressive-like behaviors and on gene expression in mood-relevant brain regions (Fig. 1a). At ~12-weeks old, mice were GDX to remove endogenous sources of gonadal hormones and to ensure equivalent gonadal hormone exposure during testing. While this approach does not allow us to assess contributions of circulating hormones, it is necessary to isolate potential effects of developmental gonadal sex, which was our focus here. In behavior cohorts, we assessed baseline anhedonia-/depressive-like behaviors [3 weeks after GDX; novelty-suppressed feeding (NSF; *n* = 23 XX Females; *n* = 20 XY Males; 22 XY Female; 16 XX Males), and social conditioned place preference (social CPP; *n* = 21 XX Females; *n* = 19 XY Males; 20 XY Females; 15 XX Males); 2 days rest between behavioral tests], exposed mice to 8 weeks of unpredictable chronic mild stress (UCMS), and then assessed stress-induced anhedonia-/depressive-like behaviors. Behaviors were examined ~3–6 h after lights on, with genotypes evenly distributed across test days. For the gene expression cohort, half the mice were exposed to 8 weeks UCMS and half were left unstressed (*n* = 9 per FCG genotype per stress group, *n* = 72 mice total); we sacrificed the mice, isolated tissue punches of the PFC, BLA, and NAc, and performed RNA-seq. Sample sizes were chosen based on previous publications^17,24^. Experimenters were blinded to genotype and stress group throughout the experiments and testing order was counterbalanced across cohorts. Animal use was conducted in accordance with the National Institute of Health guidelines for the care and use of laboratory animals and approved by the Institutional Animal Care and Use Committee of the University of Pittsburgh.

### Unpredictable chronic mild stress

We used UCMS to induce an elevated anxiety-/depressive-like state in our mice, as performed previously in our lab^17,25,26^. UCMS elicits homologous features associated with human depression. Mice were exposed to a random schedule of mild psychosocial stressors over the course of 8 weeks. Stressors included reduced space, predator odor, 45° tilted cage, social stress (exposure to dirty bedding from unfamiliar mice of the same sex), wet or no bedding, mild restraint in 50 ml conical tube with air holes, forced bath (~4 cm of 25° water), and light cycle disruption. We assessed weight and fur weekly to track progression of the UCMS syndrome. A detailed schedule of UCMS is provided in Table S1.

### Novelty-suppressed feeding

Mice were food restricted overnight and allowed to habituate to the testing room for 1 h prior to testing. Mice were then placed into the corner of a 50.8 cm × 50.8 cm novel arena, and the latency to eat a food pellet at the brightly lit (750 lux) center of the arena was recorded (12-min maximum). After testing, we recorded homecage food consumption, which did not differ between groups under non-stress (*p* > 0.15) or stress conditions (*p* > 0.35).

### Social conditioned place preference

Social CPP was performed similar to^27^. The premise is that mice prefer bedding in which they were previously socially housed versus bedding in which they were singly housed. On day 1, mice were allowed to freely explore an arena with two equally sized chambers (40 cm × 20 cm × 20 cm) for 30 min. Each chamber contained a novel bedding and time in each chamber was recorded using EthoVision (Noldus). On day 2, mice were socially housed with their cage-mates with the bedding that they spent the least amount of time in during day 1 (biased design). On day 3, mice were singly housed with the bedding they spent the most amount of time in during day 1. On day 4, mice were allowed to freely explore the testing arena containing each novel bedding and amount of time in each chamber was recorded. The social CPP score was calculated as the time spent in the social bedding post-conditioning minus the time spent in social bedding pre-conditioning. Six mice were excluded because they were socially housed in their preferred bedding during pre- or post-stress testing (pre-stress: 2 XX Females, 1 XY Female, 1 XY Male excluded; post-stress: 2 XX Females excluded). The pre-stress novel beddings were ALPHA-Dri**®** (Shepherd Specialty Papers) and Bed O’Cobs (Andersons Lab Bedding). The post-stress novel beddings were Aspen Shavings (Northeastern Products) and Paperchip**®** Soft Texture (Shepherd Specialty Papers); these are distinct from the pre-stress beddings to maintain novelty^27^.

### RNA-seq

Brains were flash frozen on dry ice at sacrifice and stored at −80 °C. 160-µm-thick rostro-caudal sections were obtained using a cryostat and a 1 mm bore tissue punch was used to isolate the prefrontal cortex (PFC; includes cingulate and prelimbic cortices; Bregma +2.34 mm to +0.50 mm), NAc (Bregma +0.74 mm to +0.38 mm), and BLA (Bregma −0.94 mm to −1.82 mm)^28^. RNA was extracted using RNeasy Plus Micro kits with Qiashredders (Qiagen). RNA integrity was assessed using a Bioanalyzer (Agilent) and concentration was determined using a Qubit fluorometer (Invitrogen). We pooled RNA from three mice for each RNA-seq run, with three biological replicates per group (3 replicates × 4 genotypes × 3 brain regions × 2 stress conditions = 72 samples). Libraries were constructed using TruSeq Stranded mRNA (PolyA+) and sequenced by Illumina NextSeq 500. All samples were sequenced across all lanes to avoid lane bias by a technician blind to treatment groups (single-end). The read length was 75 base pairs. Average sample coverage was 30.72 million reads before alignment. FastQC (v0.11.3) was performed to assess the quality of the data. Per base sequence quality was high (Phred score generally >30), indicating good quality of the experimental data. TopHat2 (ref. ^29^) (TopHat v2.0.9) was used to align the read to the reference (*Mus musculus* UCSC mm10, downloaded at https://support.illumina.com/sequencing/sequencing_software/igenome.html) using default parameters. The resulting bam files after alignment were converted to expression count data using HTseq^30^ (HTseq v0.6.1) with default union mode. A total of 72 samples (24 BLA, 24 FC, and 24 NAc) with 24,421 genes were preprocessed together. Low expression genes (25% of lower expressed genes with smallest sum of counts for all 72 samples) were filtered out and 18,337 genes remained. The RNA-seq count data were transformed to log2 count data using voom function of the Bioconductor limma package^31,32^.

### qPCR

We validated a subset of genes identified with RNA-seq using qPCR. Fifteen microliters of RNA per sample was converted into cDNA using the iScript cDNA Synthesis kit (Biorad, Hercules, CA) and quantities of cDNA were normalized across samples. qPCR was run on a CFX 96 Real-Time PCR (Biorad, Hercules, CA) using SsoAdvanced Universal SYBR Green Supermix (Biorad, Hercules, CA). We ran each gene and sample combination in triplicate and derived the mean of the replicates. Results were then calculated as the geometric mean of the relative intensities compared to two housekeeping genes, cyclophilin and actin. Importantly these housekeeping genes did not differ by sex or stress. We then transformed this value to the arbitrary signal using the formula (2^−dCT^ × 10,000). Primer efficiency and specificity were verified; primer sequences are included in Table S2.

### Data analysis

For behavioral analyses, we used two-way repeated measures (pre- vs. post-UCMS) ANOVA with 4 groups (XX Female, XY Female, XX Male, XY Male); significant group effects were followed by planned two-group comparisons (Fisher’s LSD for effects of genotype based on pairwise comparisons between group means—e.g., XX Female vs. XY Male, XX Female vs. XY Female, XX Male vs. XY Male, etc.; and Sidak’s multiple comparison test for within genotype effect of stress—e.g., Pre-UCMS vs. Post-UCMS XX Female, XY Male, etc.). All behavioral data are expressed as mean ± SEM and statistical significance was set at *p* < 0.05. Trend level was set at *p* < 0.1. Genes with *p* < 0.05 and fold change >1.3 were considered differentially expressed (DE). INGENUITY**®** Pathway Analysis (Qiagen) was used to determine canonical pathways represented by DE genes as follows: (1) 18,321 annotated genes remaining after filtering were used as background data set; (2) IPA pathways with <20 genes were not included. We used rank–rank hypergeometric overlap (RRHO) as a threshold-free method to evaluate the overlap of differential expression patterns across pairs of brain regions^33,34^. RRHO identifies overlap between two ranked lists of differential gene expression. The genes are ranked by the −log_10_(*p* value) multiplied by the effect size direction. Each brain region from all stressed mice were analyzed together through weighted gene co-expression network analysis (WGCNA)^35,36^. Module preservation analysis was performed to determine whether the modules identified were robust^37^. Analysis was based on the module size and compared to an independent network, where *Z*-scores measure whether a given module is significantly robust relative to a random set of genes. *Z*-scores were calculated for each module and ranked accordingly; summary *Z*-scores more than 10 are considered highly preserved, and these are the only modules included in downstream analyses. The module differential connectivity (MDC) metric was used to quantify differences in co-expression between non-stressed and stressed samples; MDC is the ratio of the connectivity of all gene pairs in a module from stressed mice to that of the same gene pairs from non-stressed mice^38^. To compare module connectivity between stressed and non-stressed mice, subsets of adjacent matrices were generated for sex and stress or non-stress mice, then directly compared using the ratio of summed lower triangular values. These summed lower triangular values represent the MDC metric. Connectivity is permuted to derive *p* values by randomizing values across the lower triangular matrix. Genes within each module are also permuted, and the *p* value for the MDC is compared to the null distribution to determine modules which were gained or lost between stressed and non-stressed mice. MDC > 1 indicates gain of connectivity, while MDC < 1 indicates loss of connectivity. To statistically test the significance of MDC, we estimated the *p* value based on two types of shuffling schemes: (1) shuffled samples—adjacent matrix with non-random nodes but random connections; (2) shuffled genes—adjacent matrix with random nodes but non-random connections. Data were permuted 1000 times. *P* values were converted to *q* values following Benjamini Hochberg procedure, and MDC *q* < 0.05 was considered significant. ARACNe was used to identify hub and stress-specific hub genes for network analysis^39^. Briefly, a gene is considered a hub if the N-hob neighborhood nodes (NHNN) for that gene is significantly higher than the average NHNN. A hub gene is considered a stress-specific hub if it attains hub status only in the chronic stress condition. Cytoscape 3.5.0 was used to generate networks, with correlations greater than 0.95 plotted in the networks.

## Results

### Sex differences in behavior are driven by both developmental gonadal sex and genetic sex

FCG mice were run through anhedonia-/depressive-like behavioral assays under non-stress conditions, and then exposed to UCMS and re-tested on the same behavioral assays. Since mice were GDX in adulthood, any gonadal sex differences are considered the organizational effects of hormones acting during sensitive developmental periods. In the NSF, the two-way repeated measures ANOVA indicated a main effect of genotype (*F*(3, 77) = 3.806; *p* < 0.05) and an effect of time (pre- vs. post-stress; *F*(1, 77) = 13.83; *p* < 0.001; Fig. 1b). We first established that we could detect a sex difference in latency to eat by comparing “pure” females (XX Females; genetic and gonadal females) to “pure” males (XY Male; genetic and gonadal males). Indeed, XX Females had a longer latency to eat compared to XY Males (XX Females vs. XY Males; *t*(77) = 2.207; *p* < 0.05). We next asked whether this sex difference was due to an effect of gonadal or genetic sex. We find that the sex difference in latency to eat was recapitulated in both XX Males (XX Males vs. XX Females; *t*(77) = 2.97; *p* < 0.01) and XY Females (XY Females vs. XX Females; *t*(77) = 2.207; *p* < 0.01); this suggests that both developmental gonadal sex and genetic sex contribute to the sex difference in latency to eat in NSF. In the social CPP test of social reward, the two-way repeated measures ANOVA indicated an effect of genotype (*F*(3, 72) = 7.511); *p* < 0.001) and an interaction of genotype and time (pre- vs. post-stress; *F*(3, 72) = 4.543; *p* < 0.01; Fig.1c). We confirmed that there was a sex difference in social CPP score (XX Females vs. XY Males; *t*(72) = 3.294; *p* < 0.01) by comparing pure females (XX Females) to pure males (XY Males). This sex difference in social CPP score was recapitulated in XX Males (XX Males vs. XX Females; *t*(4.17) = 4.17; *p* < 0.0001), but not in XY Females (*p* > 0.25 vs XX Females). Thus, the sex difference in social CPP is driven by developmental gonadal sex. Post hoc analyses for the significant interaction of genotype and time (pre- vs. post-stress) indicated an effect of stress only in XX Males (*t*(72) = 3.046; *p* < 0.01).

### Gonadal and genetic sex differences in gene expression under non-stress conditions

**Fig. 2:**
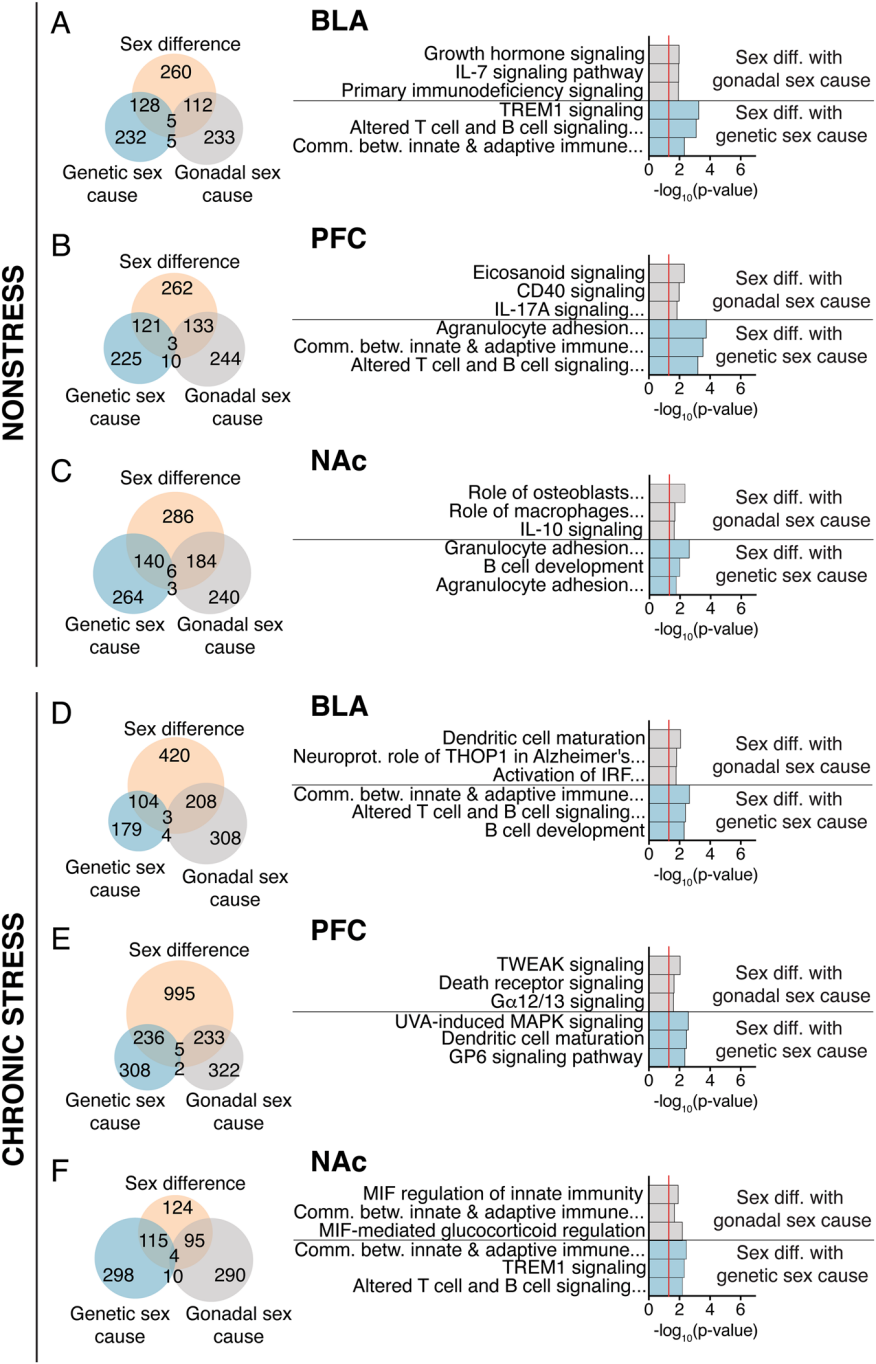
Differential gene expression reveals sex differences in genes related to immune function. Venn diagrams indicating overlap of genes exhibiting sex differences (XX Females vs. XY Males), genes that are differentially expressed based on gonadal sex (females vs. males), and genes that are differentially expressed based on genetic sex (XX vs. XY). Venn diagrams as well as top biological pathways are shown for the BLA non-stress (**a**), PFC non-stress (**b**), NAc non-stress (**c**), BLA stress (**d**), PFC stress (**e**), and NAc stress (**f**).

To identify potential molecular drivers related to sex differences in behavior, we performed RNA-seq in a separate cohort of mice. First, we identified genes with overall sex differences in expression by comparing XX Females and XY Males; there were 505 DE genes in BLA, 516 DE genes in PFC, and 610 DE genes in NAc (Fig. 2a–c; Table S3). We next investigated whether these sex differences were driven by either gonadal sex or genetic sex. For all three brain regions, roughly 25% of sex differences in gene expression were driven by genetic sex (Table S4) and 23–31% (BLA-NAc; Table S5) were driven by developmental gonadal sex. As expected, we observed that sex differences in X and Y chromosome genes were driven by genetic sex (e.g., *Ddx3y*, *Eif2s3x*, *Kdm5d*, *Xist*). We also found sex differences in expression of many activity-dependent genes (e.g., *Arc*, *Fos*, *Egr2*, *Npas4*); these sex differences were driven by developmental gonadal sex and more highly expressed in gonadal females. Although the exact genes driven by genetic or gonadal sex differ, pathway analysis revealed that the genes driven by genetic sex and gonadal sex typically involve immune function and inflammation (Fig. 2a–c). We validated a subset of genetic and gonadal sex differences in gene expression under non-stress conditions using qPCR (Fig. S1A; Table S12); 63% of genes were significantly different by qPCR and all showed the same direction of effect as RNA-seq.

### Gonadal and genetic sex differences in gene expression under chronic stress conditions

We also investigated sex differences in gene expression under chronic stress conditions. We first identified genes with overall sex differences by comparing XX Females and XY Males; there were 732 DE genes in BLA, 1464 DE genes in PFC, and 338 DE genes in NAc (Fig. 2d–f; Table S6). We next asked if gonadal sex or genetic sex drives any of these observed sex differences in gene expression. Overall, the contribution of genetic sex to these differences were lower relative to non-stress conditions: 14% and 16% for BLA and PFC, respectively, with 35% in the NAc (Fig. 2d–f; Table S7). Gonadal sex accounted for 28% in BLA, 16% in PFC, and 29% in NAc (Fig. 2d–f; Table S8). As predicted, across brain regions, genetic sex drives sex differences in X and Y chromosome gene expression. Notably, none of the activity-dependent genes that were DE under non-stress conditions were DE in any brain region under chronic stress. Pathway analysis revealed that the genes driven by genetic sex and gonadal sex typically involve immune function and inflammation (Fig. 2a–c). We validated a subset of genetic and gonadal sex differences in gene expression under stress conditions using qPCR (Fig. S1B); 50% of genes were significantly different by qPCR and all showed the same direction of effect as RNA-seq.

### Sex-specific transcriptional effects of chronic stress

**Fig. 3:**
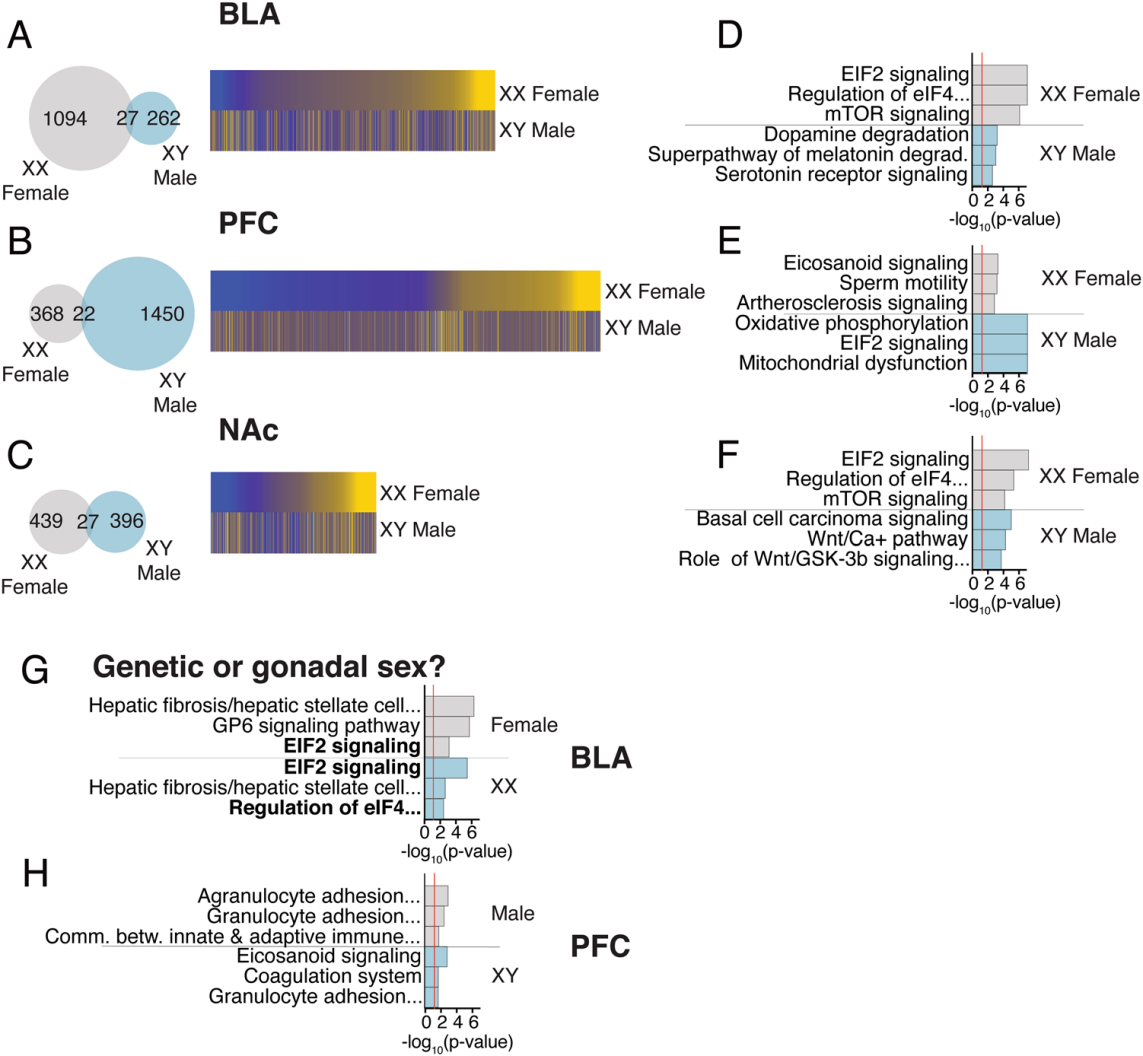
Sex differences in genes and pathways affected by stress. **a**–**c** Venn diagrams and heatmaps indicating overlap in genes affected by stress in XX Females and XY Males across brain regions. **d–f** Pathways affected by stress in XX Females and XY Males across brain regions. **g** Since the strongest effect of stress in XX Females was in the BLA, we next used the FCG mice to determine whether this effect of stress was driven by genetic or gonadal sex. Pathways affected by stress in the BLA of gonadal females and XX mice, which indicate that both gonadal sex and genetic sex contribute to the effect of stress in the BLA of females. **h** Since the strongest effect of stress in XY Males was in the PFC, we next used the FCG mice to determine whether this effect of stress was driven by genetic or gonadal sex. Pathways affected by stress in the PFC of gonadal males and XY mice. The contribution of gonadal sex or genetic sex to the effect of stress in the PFC was less pronounced and indistinguishable between factors.

We examined stress-induced transcriptional changes to investigate whether there are sex-specific effects of stress on genes in each brain region. Notably, our behavioral analyses suggest that the chronic stress exposure impacted males and females similarly (i.e., increased anhedonia-/depressive-like behavior after chronic stress). While males and females arrive at the same behavioral endpoint, we asked whether similar molecular changes underlie these stress-induced effects in males and females. A direct comparison of stress DE genes in XX Females and XY Males revealed just 1–10% overlap across the three brain regions (Fig. 3a–c; Table S9). The biological pathways associated with stress DE genes are also distinct in females and males (Fig. 3d–f). We next used the FCG mice to determine whether any stress-sensitive pathways are recapitulated when we examine XX mice versus XY mice (Table S10) and gonadal males versus gonadal females (Table S11). The strongest effect of stress was in the BLA of females (Fig. 3a); pathway analysis determined that both gonadal and genetic sex strongly contribute to the female effect of stress in the BLA (Fig. 3g). For males, the strongest effect of stress was in the PFC (Fig. 3b), although the contribution of gonadal sex or genetic sex to the effect of stress in the PFC was less pronounced and indistinguishable between factors (Fig. 3h). Stress-induced DE overlap and biological pathways for gonadal females versus males and XX versus XY mice are provided for all brain regions in Figs. S2 and S3; these also show overlap of stress-induced DE genes with those showing sex differences under non-stress conditions. We validated a subset of genes exhibiting effects of stress using qPCR (Fig. S1C); 64% of genes were significantly different by qPCR and all but three showed the same direction of effect as RNA-seq.

### Sex-specific transcriptional synchrony across mood-relevant brain regions

**Fig. 4:**
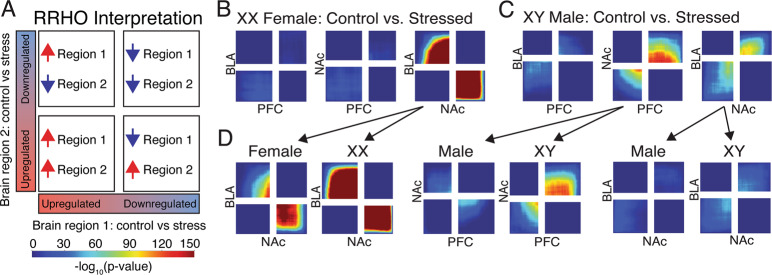
Inter-brain region differential expression patterns reveal sex-specific transcriptional signatures. **a** Schematic depicting interpretation for RRHO plots. A hot spot in the bottom left corner indicates overlap in genes upregulated by stress in both brain regions. A hot spot in the top right corner indicates overlap in genes downregulated by stress in both brain regions. A hot spot in the top left indicates overlap in genes upregulated in brain region 1 and downregulated in brain region 2. A hot spot in the bottom right indicates overlap in genes downregulated in brain region 1 and upregulated in brain region 2. **b** In XX Females, there was overlap of genes affected by stress in the opposite direction between the NAc and BLA (i.e., increased by stress in the BLA, decreased by stress in the NAc, and vice versa). **c** In XY Males, there was transcriptional overlap in genes affected similarly by stress between the PFC and NAc as well as between the NAc and BLA. **d** The opposite effect of stress on gene expression between the NAc and BLA of XY Females is recapitulated in both gonadal females and in XX mice. The effect of stress on gene expression between the PFC and NAc of XY Males is recapitulated in only XY mice.

Since mood disorders often involve circuit-level changes across multiple brain regions, we next examined transcriptional coherence across brain regions in response to stress. We used the RRHO test to identify patterns of gene expression changes in response to stress^33,34^. RRHO identifies patterns of upregulated and downregulated genes without the restriction of significance thresholds. We used RRHO to investigate sex-specific patterns of cross-brain region transcriptional synchrony in response to stress. RRHO identifies four possible patterns: (1) overlap in genes that are increased by stress in both regions (coherence); (2) overlap in genes that are decreased by stress in both regions (coherence); (3) overlap in genes that are increased in region 1, but decreased in region 2 (anti-coherence); (4) overlap in genes that are decreased in region 1, but increased in region 2 (anti-coherence; Fig. 4a). RRHO was used previously to investigate transcriptional coherence in mood-related brain regions associated with stress susceptibility and resilience in mice, which revealed molecular mechanisms critical for depression-like behaviors^40^. We first examined stress-induced transcriptional coherence between brain regions in XX Females and XY Males. In XX Females, we found strong transcriptional anti-coherence between the NAc and BLA. In other words, in XX Females, stress induced opposite changes in expression of the same genes in the BLA and NAc (Fig. 4b); this pattern did not exist in XY Males. Rather, XY Males showed a weak pattern of coherence between the NAc and BLA (Fig. 4c). Additionally, in XY Males, there was coherence between the PFC and NAc; this pattern did not exist in XX Females. We next asked whether these cross-brain region transcriptional coherence patterns are recapitulated by gonadal or genetic sex. For the anti-coherence between the BLA and NAc observed for XX Females, this pattern is recapitulated in both gonadal females and XX mice (Fig. 4d). For the coherence between the BLA and NAc observed in XY Males, the pattern is weakly recapitulated in both gonadal males and in XY mice (Fig. 4d). For the coherence between the NAc and PFC in XY Males, the pattern is recapitulated in only XY mice.

### Co-expression analysis identifies gene modules that are affected by stress in a sex-specific manner

**Fig. 5:**
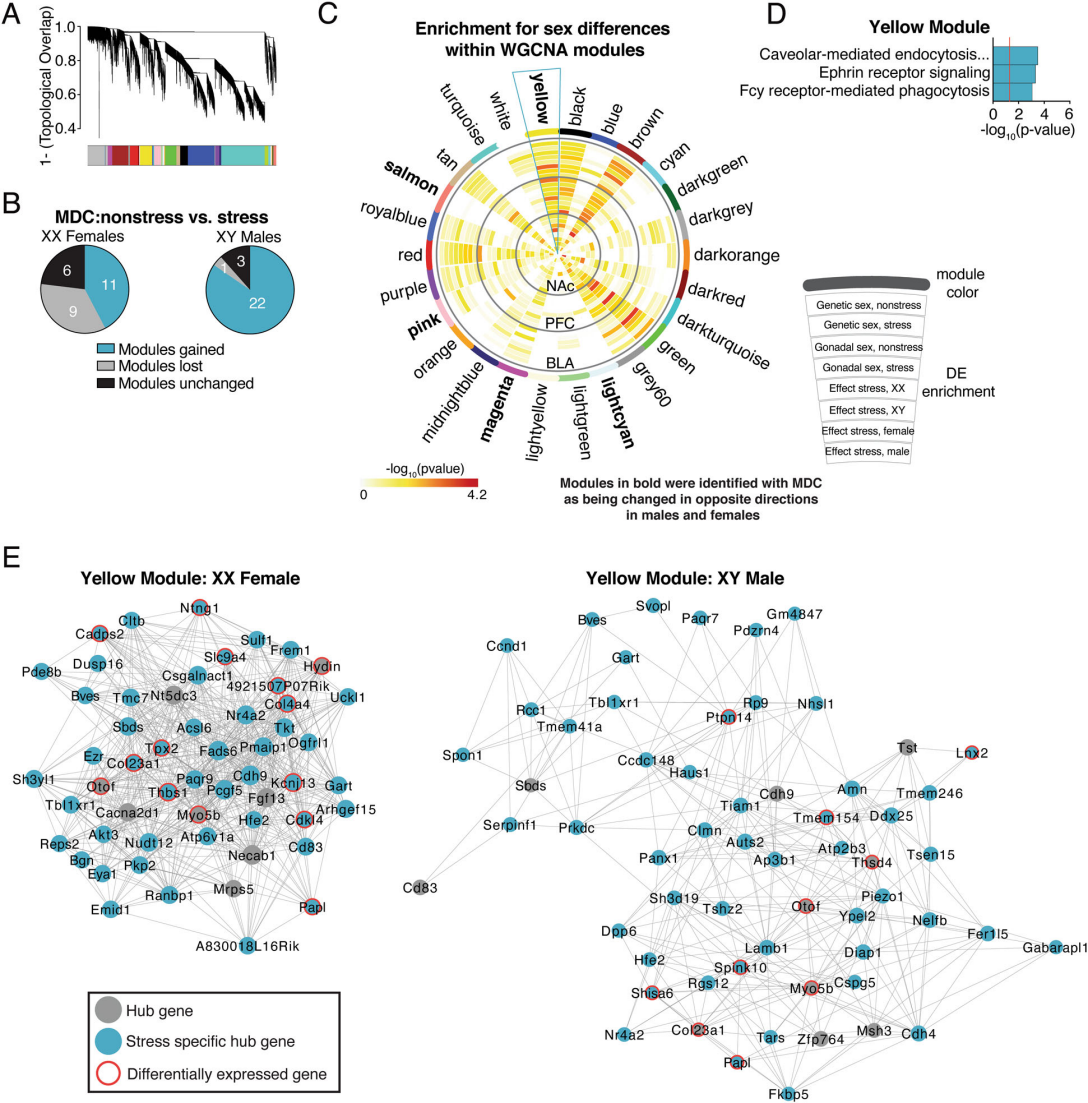
Identification of co-expression networks that are differentially affected by stress in males and females. **a** Weighted gene co-expression network analysis (WGCNA) was used to generate co-expression modules, with the network structure generated using all stressed samples from all three brain regions. The 26 identified modules that survived module preservation analysis were arbitrarily assigned colors and the dendrogram shows average linkage hierarchical clustering of genes. **b** Pie charts summarize results from the module differential connectivity (MDC) analysis. Many more modules were gained in XY Males compared to XX Females. **c** Circos plot for all 26 WGCNA modules. Module names and colors are indicated on the outside of the circos plot. Enrichment for various differential expression analyses are indicated by the semi-circle colors within each module, with increasing warm colors indicating increasing −log_10_(*p* value). The yellow module was particularly interesting, as the MDC analysis indicated that stress induced a loss of connectivity of this module in XY Males, but a gain in connectivity in XX Females. Additionally, this module showed enrichment for many sex differences. **d** Pathway analysis for the yellow module. **e** Hub gene co-expression networks of the yellow module in XX Females and in XY Males. Node size indicates the degree for that gene. Turquoise nodes indicate stress-specific hub genes, gray nodes indicate hub genes, and red halos indicate differential expression for that gene. Edges indicate significant co-expression between two particular genes.

We next identified gene modules differentially affected by stress in males and females. First, we generated a gene co-expression network using all stressed samples from all brain regions; this stressed network consisted of 30 gene modules, with 26 modules surviving preservation analysis (Fig. 5a). Given our focus on potential sex-specific effects of stress, we next used module differential connectivity (MDC) to ask whether module connectivity increased or decreased as a function of stress; this analysis was performed separately in XX Females and XY Males. If a module gains connectivity as a function of stress, it exhibits more coordinated expression in stressed compared to non-stressed mice. On the other hand, a module that loses connectivity as a function of stress is more highly coordinated in non-stressed than in stressed mice. In XX Females, 11 modules (42%) gained connectivity, 9 modules (35%) lost connectivity, and 6 modules (23%) did not change as a function of stress. In XY Males, 22 modules (85%) gained connectivity, 1 module lost connectivity (4%), and 3 modules (12%) did not change with stress exposure (Fig. 5b).

We recently found that depressed men and women exhibit opposite patterns of transcriptional changes across corticolimbic brain regions^41^. Thus, we were particularly interested in gene modules which were lost or gained in opposing directions based on sex in chronically stressed FCG mice. We asked if stress exposure induced gain of module connectivity in one sex, but loss of connectivity in the opposite sex. Indeed, there were five modules that gained connectivity in XY Males, but lost connectivity in XX Females (yellow, salmon, pink, magenta, and lightcyan). The yellow module was particularly interesting, as it showed enrichment for differentially expressed genes (Fig. 5c). This module was enriched in genes related to Caveolar-mediated endocytosis, Ephrin receptor signaling, and Fcγ receptor mediated phagocytosis (Fig. 5d). We decided to examine the yellow module in more detail using network analysis, depicting only “hub genes” for XX Females or XY Males. Based on their high level of co-expression within the module, hub genes are predicted to control expression of other genes within the module. “Stress-specific hub genes” are predicted to control expression of other genes within the module in stressed mice, but not under non-stress conditions; these stress-specific hubs might be key upstream regulators driving divergent sex-dependent stress effects. The composition and topography of the yellow hub network is markedly different in XX Females and XY Males (Fig. 5e). Not only is the network much more correlated in XX Females than XY Males, but many of the hub genes are distinct (overlap of ~20% of hub genes). This is consistent with the yellow module gaining connectivity in stressed XX Females, but losing connectivity in stressed XY Males (see MDC results). Examination of the stress-specific hub genes in this network provides key leads for potential upstream drivers of the sex-specific response to stress. For instance, the yellow module has 11 stress-specific hub genes in XX Females and 6 stress-specific hub genes in XY Males; *Papl* is the only gene that is a stress-specific hub in both XX Females and XY Males. Interestingly, the *Papl* gene is located within an anxiety quantitative trait loci (QTL) as well as a neuroinflammation QTL^42,43^. Two of the stress-specific hub genes in XX Females are related to synapse development (*Thbs1* and *Cadps2*), which is notable given previous evidence for synapse dysfunction in MDD, with potential sex specificity^41,44^.

## Discussion

Recent studies in the human brain suggest the molecular pathology of depression is distinct in men and women^41,45^, although the contribution of gonadal and/or genetic sex to these overall sex differences is less clear. We found that sex differences in anhedonia-/depressive-like behavior are driven by both developmental gonadal hormone exposure (i.e., gonadal sex) and genetic sex. Our transcriptome analysis revealed gonadal and genetic sex differences in mood-relevant brain regions, providing molecular leads that may drive sex differences in behavior. Further, we found distinct brain molecular profiles in males and females exposed to chronic stress. This finding is consistent with the human literature of distinct molecular pathology in depressed men and women and suggests contributions of both genetic and gonadal sex.

Since depression is known to involve circuit-level disruption, examining the global impact of stress on molecular pathways within these circuits in the brain is informative. Indeed, our results suggest that stress impacts circuit-level mesocorticolimbic function in a sex-specific fashion, with strong anti-coherence of stress-induced transcriptional changes between the NAc and BLA in XX Females, a pattern not found in XY Males. Rather, XY Males showed synchrony between the NAc and PFC. The source of these alterations is unclear. For example, these may be due to stress causing sex-dependent changes in the functional connections between or within these regions (e.g., neural activity, cellular and synaptic plasticity), or alterations in the activity of upstream molecular regulators of overlapping molecular signaling pathways^40,46–50^. Another possibility is that transcriptional coherence between brain regions reflects resting state activity patterns, although this has not been experimentally tested. Indeed, depressed subjects exhibit changes in resting state connectivity. Patients with an anxious subtype of MDD have decreased frontoamygdala connectivity, while patients with an anhedonia subtype have frontostriatal hyperconnectivity^51^. Interestingly, we see that females have reduced coherence between the PFC and BLA compared to males (possibly similar to reduced frontoamygdala connectivity in MDD), and that XY Males have increased coherence between the PFC and NAc (possibly similar to hyperactivity in frontostriatal network in MDD). Together, these findings suggest that stress produces sex-specific changes in mesocorticolimbic circuitry and provide novel circuit-based hypotheses to test in future studies.

The identified sex differences in gene expression provide molecular leads for pathways which may underlie observed sex differences in behavior. Many biological pathways represented by these genes are related to immune function; this is true for sex differences within non-stress or stress conditions, effects of stress on gene expression, as well as for the identified yellow module that shows a sex-specific effect of stress. This is especially interesting given the immune/inflammatory hypothesis of MDD, which posits that elevated peripheral inflammation contributes to the pathophysiology of depression. Reports show that MDD subjects have altered peripheral cytokine profiles (e.g. refs. ^52,53^), and there is high co-morbidity between immune disorders and MDD^54^. The story, however, is complicated, with evidence for both pro-inflammatory^55^ and immunosuppressive profiles in MDD^52,53^. One factor that might contribute to this heterogeneity is sex. For instance, male MDD seems to be characterized by inflammation-related immune profile not seen in depressed females (e.g., ref. ^56^). There are also reported sex differences in peripheral immune markers^57,58^. Investigation of peripheral pro-inflammatory cytokines in depressed subjects showed sex differences in correlation of these markers and MDD severity^58^. Similar sex-specific findings were reported for healthy subjects given a peripheral immune challenge; females experienced more depression-related symptoms, and there was a positive correlation of pro-inflammatory interleukin-6 (IL-6) levels and depression-related symptoms only in females^59,60^. Together, this evidence suggests that intrinsic sex-related factors might interact with immune function to influence mood.

Under non-stress conditions, there were consistent and robust gonadal sex differences in expression of immediate early genes (IEGs), including *Arc, fos, Egr1*, and *Npas4*, with gonadal females having higher expression than gonadal males. Prior to tissue collection, these mice were handled similarly; notably, there was no acute stress exposure, except for the short transport of their cages from holding to procedure rooms. Since IEG expression is considered a marker of neuronal activation, one possibility is there is an inherent sex difference in neuronal activity, which is dependent on developmental gonadal hormones. Basal patterns of neuronal activity are linked to differential waves of activity-dependent gene transcription involving IEGs^61^. For example, *Egr1* has relatively high basal expression across the brain, which is thought to be in response to normal neural activity^62–65^. Further, many of these IEGs act as transcription factors to activate molecular pathways involved in neural plasticity in response to various stimuli^66,67^, including drugs of abuse and stress^68–70^, and previously linked to anhedonia, a core feature of MDD and addiction disorders. Notably, altered *EGR1* has been found in the PFC of MDD patients^71^ and in other brain regions in animal models of stress-induced depressive-like behavior^72–76^. *Npas4* is another IEG of interest and may be a key driver of excitatory and inhibitory balance^77^, potentially dependent on sex and also affected by stress^78,79^. Another intriguing possibility is the upregulation of these IEGs in gonadal females may be a result of a more permissive, stable chromatin state. Indeed, sex differences in chromatin accessibility and transcription are found in other tissues^80,81^.

The WGCNA and MDC analyses suggest that several gene modules are oppositely affected by stress in XX Females and XY Males. This result was particularly intriguing to us given our recent finding of opposite changes in gene expression in the brains of depressed men and women^41^. Thus, we decided to focus on these oppositely affected modules for downstream analyses. When examining enrichment for sex differences within these oppositely affected modules, the yellow module stood out as being enriched for sex differences across mesocorticolimbic brain regions. The top pathway represented by the genes in the yellow module was related to caveolar-mediated endocytosis. This is notable given that caveolin proteins traffic and cluster receptors to the cell membrane, including D1 dopamine receptors, M1 muscarinic receptors, and estrogen receptors^82–84^. Interestingly, a recent study identified estrogen receptor alpha (ERα) in the NAc as a top transcriptional regulator of stress resilience, with higher ERα conferring resilience^85^. This study also found that ERα overexpression in the NAc reproduced the transcriptional signature consistent with resilience in male, but not female mice. Whether these stress-resilience properties are mediated via intracellular or membrane-bound ERα (which would rely on caveolar-mediated processes) remain to be determined.

The gene co-expression network analysis of the yellow module identified several genes that might drive sex differences in response to stress. For instance, within the yellow module, there are two synaptic-related genes (*Thbs1* and *Cadps2*) that are stress-specific hubs in XX Females, but not XY Males; these genes are also differentially expressed by sex. Notably, synaptic dysfunction has been implicated in MDD. There are reduced dendritic processes/spine synapses in the dorsolateral prefrontal cortex in MDD^44^. Chronic stress in male rodents recapitulates this dendritic pathology^86,87^, and it is hypothesized that dendritic spine alterations contribute to MDD symptoms^88,89^. However, we found opposite gene expression changes in MDD females, with increased expression of synapse-related genes^90^. Our results in males and females with MDD are consistent with rodent studies in which stressed males have decreased dendritic spine complexity, but exactly the opposite occurs in stressed females^86,91^. Together, our network analysis points towards potential upstream regulators of these sex-specific disease/stress effects on synapses.

It is important to note that these behavioral tests were developed in male mice; thus, it is possible that these specific tests do not tap into mood-related behavior in females. For instance, it is possible that females exhibit either different motivation for food in the NSF or differences in learning in the social CPP. However, we note that XY Females and XX Males respond similarly to XY Males, suggesting that these behavior tests “work” in both sexes. Additionally, it is possible that more than one pairing in each social context may be necessary for social CPP. We based our social CPP design on previously published papers^27,92,93^. For instance, using a similar design, Nardou et al.^93^ reported robust social conditioning in mice which required no additional social pairings. Here, we observed social conditioning prior to stress exposure that was largely lost following stress. However, we acknowledge that including additional conditioning days could have improved the level of social conditioning post-stress. In any case, sex differences in the behavioral tests assessed here exist, and they are driven by both developmental gonadal sex and genetic sex. Notably, all mice used in these studies underwent GDX several weeks prior to behavioral testing, stress exposure, and/or gene expression analyses. GDX may impact the peripheral stress response (i.e., corticosterone), which may have downstream effects on molecular/behavioral outcomes. Future studies will investigate whether the sex-specific effects on behavior and gene expression are mediated by changes in corticosterone. Interestingly, Hodes et al.^94^ found sex differences in mood-related behavior after 6 days of subchronic variable stress (SCVS). It would be interesting to use FCG mice to determine whether the sex differences after SCVS are due to genetic or gonadal sex.

To conclude, our study points towards a role for both developmental hormones and genetic sex in programming adult sex differences in mood. The cross-brain region coherence analysis suggests that stress induces transcriptional synchrony between the PFC and NAc in males, but synchrony between the BLA and NAc in females; this suggests that stress affects mesocorticolimbic circuitry differently in males and females. The impact of these circuitry changes is yet to be determined. We also identified potential upstream regulators of observed sex differences, and future studies will determine their impact on stress susceptibility.

## Supplementary information

Supplemental Tables

Supplemental Material
