# Supplementary material for: Sex differences in adult mood and in stress-induced transcriptional coherence across mesocorticolimbic circuitry
Source: Transl Psychiatry. 2020 Feb 6;10:59. doi: 10.1038/s41398-020-0742-9 (PMC7026087; doi:10.1038/s41398-020-0742-9)
Supplement: Supplementary file 2 — Supplemental Material [file 41398_2020_742_MOESM2_ESM.docx]

**SUPPLEMENTAL INFORMATION**

**Developmental origin of sex differences in adult mood and in stress-induced transcriptional coherence across mesocorticolimbic circuitry**

William Paden B.S.^1,2,#^, Kelly Barko B.S.^1,2,#^, Rachel Puralewski B.S.^1,2^, Kelly Cahill B.S.^3^, Zhiguang Huo Ph.D.^4^, Micah Shelton M.S.^1,2^, George Tseng Psy.D.^3,5^,

Ryan W. Logan Ph.D.^1,2,6,§^, and Marianne Seney Ph.D.^1,2,§^

^1^Department of Psychiatry, University of Pittsburgh School of Medicine, Pittsburgh, PA, USA

^2^Translational Neuroscience Program, University of Pittsburgh School of Medicine, Pittsburgh, PA, USA

^3^Department of Biostatistics, Graduate School of Public Health, University of Pittsburgh, Pittsburgh, PA 15261, USA

^4^Department of Biostatistics, University of Florida, Gainesville, FL 32611, USA

^5^Department of Computational and Systems Biology, University of Pittsburgh School of Medicine, Pittsburgh, PA 15213, USA

^6^Center for Systems Neurogenetics of Addiction, The Jackson Laboratory, Bar Harbor, ME 14609, USA

^§^Co-corresponding authors

**Supplementary Tables:** Uploaded separately as multi-worksheet Excel file

**Supplementary Figures**

**
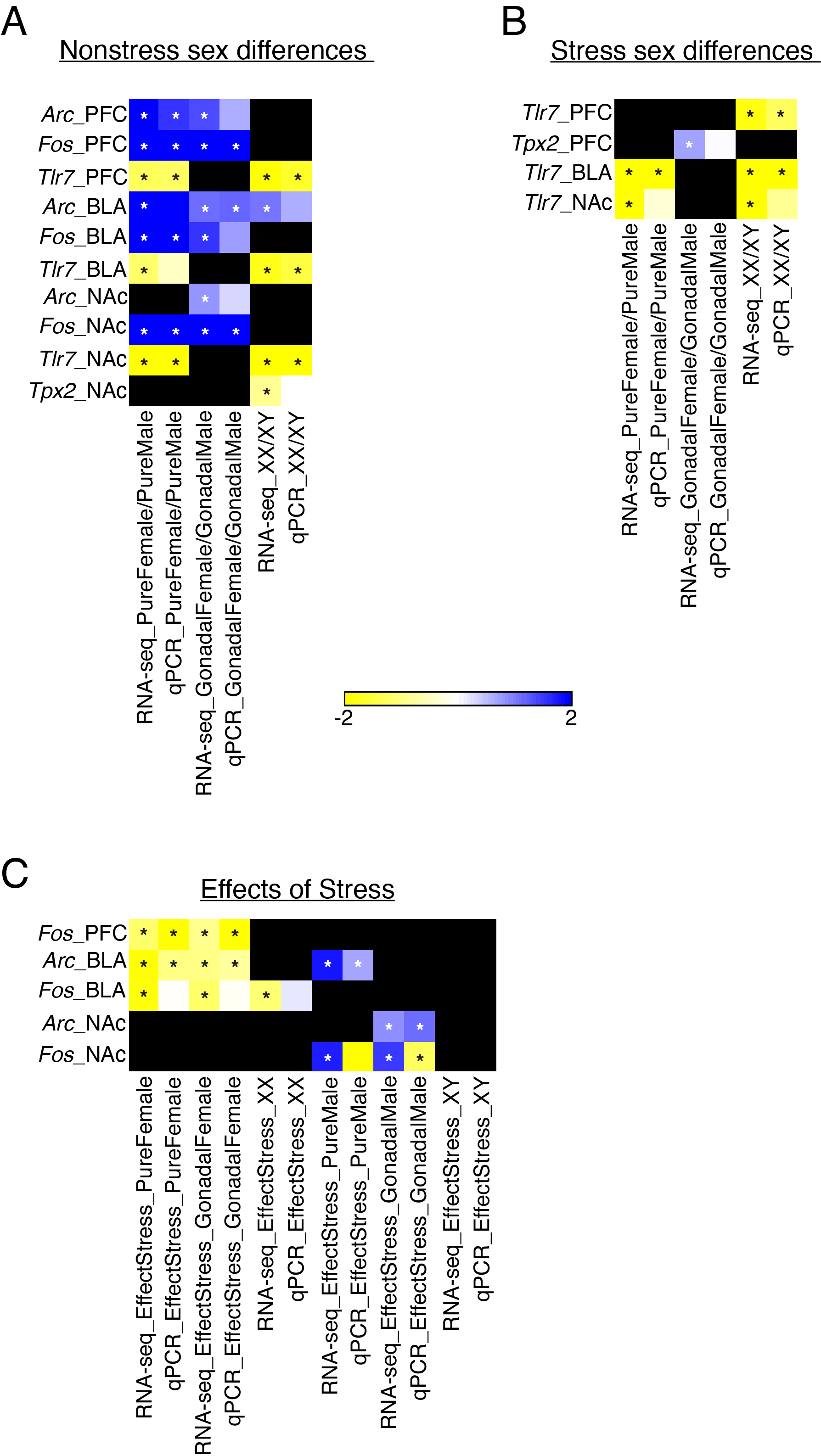
**

**Figure S1. Validation of gene expression changes.** Gene expression changes for a subset of RNA-seq hits was validated using qPCR, including genes from each region and each sex-related comparison. Expression changes are represented by fold change. Heatmaps are shown for genes exhibiting sex differences under nonstress conditions (A), sex differences under stressed conditions (B), and effects of stress within each sex (C). For RNA-seq: *, p<0.05, FC>1.3. For qPCR: *, p<0.1.

**
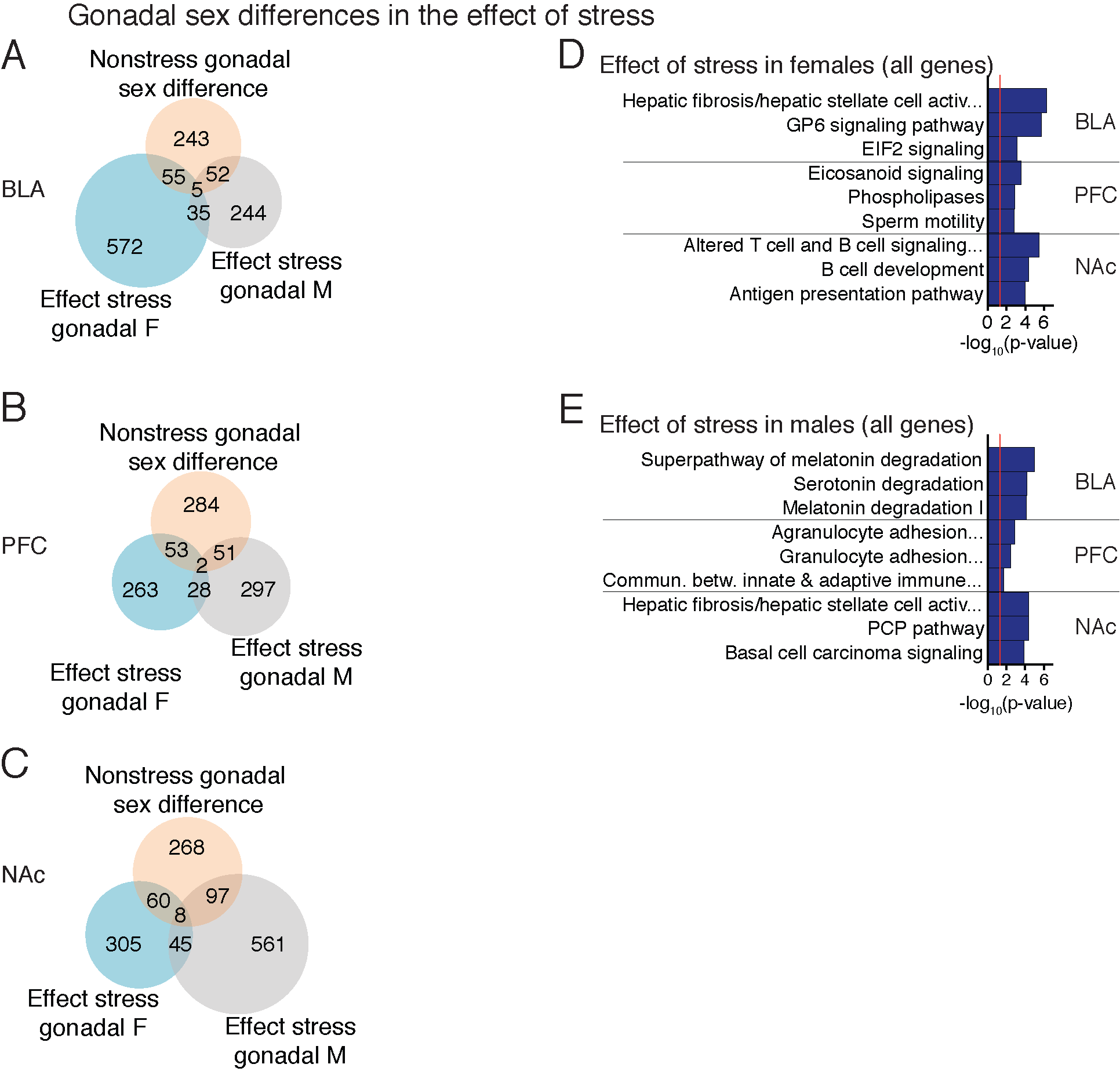
**

**Figure S2. Distinct genes and pathways affected by stress in gonadal females versus males and XX versus XY mice.** (**A-C**) A direct comparison of stress DE genes in gonadal males versus gonadal females revealed only 5-15% overlap across the 3 brain regions. We also show the overlap of these genes exhibiting an effect of stress with genes exhibiting a sex difference under nonstress condition. (**D-E**) The pathways represented by these stress-induced DE genes largely involve immune function in both males and females. We note that the exact immune-related DE genes/pathways differ between males and females. In the BLA, however, the pathways are completely distinct in males and females, with the top male/stress pathways involving melatonin and serotonin degradation, while the top female/stress pathways involving immune function and translation.


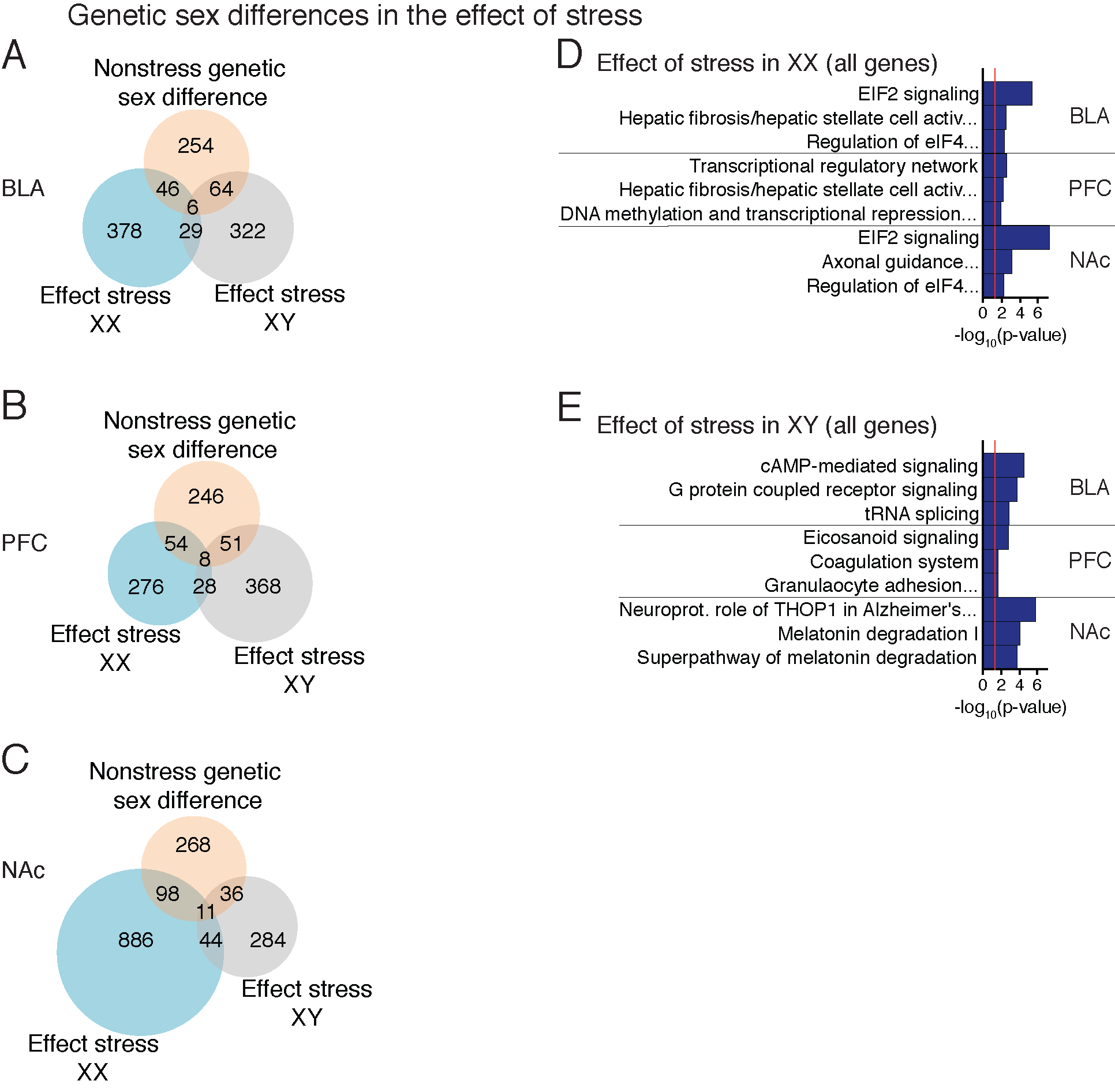


**Figure S3. Distinct genes and pathways affected by stress in gonadal females versus males and XX versus XY mice.** (**F-H**) Comparing stress-induced DE genes in XX versus XY mice revealed only 6-13% overlap across brain regions. We also show the overlap of these genes exhibiting an effect of stress with genes exhibiting a sex difference under nonstress condition. (**I-J**) We see a similar pattern of pathways affected by stress in XX/XY mice as we saw for gonadal stress, with distinct immune- and translation-related pathways in XX and XY mice. Again, the pathways affected by stress in the BLA are different in XX and XY mice; XX mice exhibit pathways related to immune function and translation, while XY mice exhibit pathways related to cAMP-mediated and G protein coupled receptor signaling.
